# Novel insights on m^6^A RNA methylation in tumorigenesis: a double-edged sword

**DOI:** 10.1186/s12943-018-0847-4

**Published:** 2018-07-21

**Authors:** Shaoyun Wang, Peiwei Chai, Ruobing Jia, Renbing Jia

**Affiliations:** Department of Ophthalmology, Ninth People’s Hospital, Shanghai Jiao Tong University School of Medicine, Shanghai, 200025 People’s Republic of China

**Keywords:** RNA methylation, m^6^A, Tumorigenesis

## Abstract

N6-methyladenosine (m^6^A), the most prevalent modification of mammalian RNA, has received increasing attention. Although m^6^A has been shown to be associated with biological activities, such as spermatogenesis modulation, cell spermatogenesis and pluripotency, *Drosophila* sex determination, and the control of T cell homeostasis and response to heat shock, little is known about its roles in cancer biology and cancer stem cells. Recent articles have noted that some genes have abnormal m^6^A expression after tumorigenesis, including genes *ABS2, RARA, MYB, MYC, ADAM19 and FOX1*. Abnormal changes in the m^6^A levels of these genes are closely related to tumour occurrence and development. In this review, we summarized the ‘dual edge weapon’ role of RNA methylation in the tumorigenesis. We discussed RNA methylation could lead to not only tumour progression but also tumour suppression. Moreover, we clarified that the abnormal changes in the m^6^A enrichment of specific loci contribute to tumour occurrence and development, thereby representing a novel anti-cancer strategy by restoration to balanced RNA methylation in tumour cells.

## Background

**Fig. 1:**
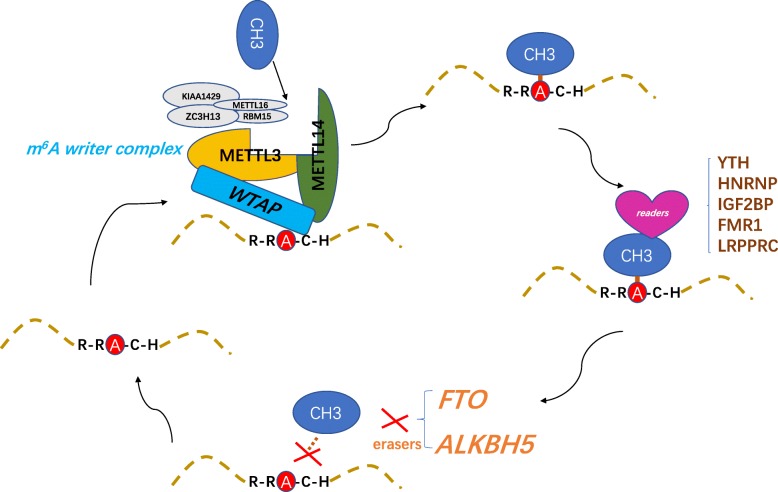
The process of m^6^A RNA modification. The installation, removal and identification of m^6^A are performed by writers, readers, and erasers, respectively. Writers interact with a special sequence of RRACH in mRNA that produces catalytic action mediated by METTL3, METTL14, KIAA1429, ZC3H13, METTL16 and WTAP. m^6^A functions are received by some reader proteins: YT521-B homology (YTH) domain-containing protein, IGF2BP, FMR1, LRPPRC and the heterogeneous nuclear ribonucleoprotein (HNRNP) protein families. Two m^6^A eraser proteins are obesity-associated protein (FTO) and alkB homologue 5 (ALKBH5)

Approximately 100 different post-transcriptional chemical modifications are present in RNA among all living organisms [1]. N6-methyladenosine (m^6^A), one such modification, was identified in the 1970s as the most abundant internal chemical modification in eukaryotic mRNA [2]. Approximately 0.1–0.4% of adenosine nucleotides in isolated mammalian RNA are chemically modified [3]. Extensive m^6^A modifications are present in the RNA of plants and vertebrates, and these modifications also occur in the RNA of single-celled organisms, such as bacteria and yeast [2, 4–7]. m^6^A-based modifications occur at a consensus motif, RRm^6^ACH([G/A/U][G > A]m^6^ AC[U > A > C]) [8] (Fig. 1). Furthermore, m^6^A is mainly concentrated on stop codons, in 3′ untranslated regions (3’UTRs), and within internal long exons, based on detection with m^6^A-specific antibodies and high-throughput sequencing [9]. A multicomponent methyltransferase complex catalysing m^6^A formation was first reported in 1994 [10]. Subsequently, methyltransferase-like 3 (METTL3), which functions as an S-adenosyl methionine-binding protein, was the first protein found to possess methyltransferase capacity [11]. Later, other m^6^A methyltransferase components were gradually discovered in mammals, including Wilms tumour 1-associated protein (WTAP), methyltransferase-like 14 (METTL14), RNA binding motif protein 15 (RBM15), KIAA1429 and zinc finger CCCH-type containing 13 (ZC3H13) (‘writers’) [12–15]. METTL3 and METTL14 form a stable complex in mammalian cells that accurately localizes at methylation sites by associating with WTAP [12]. The catalytic methylation activity of METTL14 is approximately 10 times that of METTL3, but WTAP has no catalytic methylation activity [12]. Recently, methyltransferase-like protein 16 (METTL16) was confirmed to be a m^6^A methyltransferase that methylates U6 spliceosomal RNA and interacts with the 3′-terminal RNA triple helix of metastasis-associated lung adenocarcinoma transcript 1(*MALAT1*) [16]. In 2011, the first demethylase fat mass and obesity-associated protein (FTO) was identified, demonstrating that m^6^A modifications on mRNA are reversible and dynamic [17]. FTO and alkB homologue 5 (ALKBH5) function as two kinds of demethylases (‘erasers’) and may target distinct sets of target mRNAs [18, 19]. Members of the YT521-B homology (YTH) domain family of proteins (YTHDF1, YTHDF2, YTHDF3, YTHDC1 and YTHDC2) have a conserved m^6^A-binding pocket and directly read m^6^A-mediated physiological effects [9, 20–25]. Heterogeneous nuclear ribonucleoprotein (HNRNP) proteins HNRNPA2B1 and HNRNPC selectively bind m^6^A-containing mRNAs to respond to physiological effects [26, 27]. These proteins influence mRNA processing by impacting functions such as mRNA splicing, export, and translation initiation [24, 26, 28]. Recently, insulin-like growth factor 2 mRNA-binding proteins (IGF2BPs; including IGF2BP1/2/3) were found to recognize m^6^A RNA modifications, functioning as a distinct family of m^6^A readers [29]. In addition, fragile X mental retardation 1 (FMR1) and leucine rich pentatricopeptide repeat containing (LRPPRC) read m^6^A modifications on target loci and influence RNA behaviour [30] (Fig. 1).

N6-methyladenosine represents one of the most common RNA modifications in eukaryotes, regulating RNA behaviours such as splicing or the ability to code diverse proteins [9, 20–25]. Recently, aberrant m^6^A modification in the large internal exon of a tumour suppressor was shown to give rise to premature polyadenylation, leading to membrane associated guanylate kinase (*MAGI3)* inactivation [31]. In addition, m^6^A regulates other forms of RNA modification. There is a significant negative correlation between two distinct and abundant RNA modifications, m^6^A and adenosine-to-inosine (A-to-I), suggesting a previously underappreciated interplay between them [32]. N6-methyladenosine also affects the function of Long non-coding RNA (LincRNA). *LincRNA 1281* is required for proper differentiation of mouse embryonic stem cells, and this critical function relies on sufficient m^6^A modification [33]. m^6^A may also play a functional role by regulating histones and DNA. The homeostatic regulation of S-adenosylmethionine (SAM) synthesis in mammalian cells involves dynamic m^6^A modifications on the 3’UTR of methionine adenosyltransferase 2A (*MAT2A)* [34]. In this review, we will briefly introduce physiological activities related to m^6^A modification. Then, we will describe in detail the ability of m^6^A modifications, functioning as a double-edged sword, to play a regulatory role in tumorigenesis and development.

### Physiologic functions of m^6^A modifications

The dynamic reversibility of m^6^A methylation suggests that it plays an important role in physiological processes. Studies have revealed that m^6^A modifications on mRNAs or non-coding RNAs play important roles in spermatogenesis, T cell homeostasis, *Drosophila* sex determination, heat shock responses, pluripotency and reprogramming, as well as other processes [27, 35–39] (Table 1).

**Table 1:** m^6^A chemical modifications affect physiological function

Physiological activities	Genes involved	Description	Reference
Spermatogenesis	Plzf, Dnmt3b Id4 and Sohlh2	Deletion of m^6^A results in the dysregulation of spermatogenesis	[39]
T cell homeostasis	SOCS1, SOCS3 and CISH	Decreased m^6^A modification inhibits naive T cell proliferation and differentiation but maintains cell survival	[40]
*Drosophila* sex determination	Sxl	YT521-B reads the m^6^A modification of Sxl to promote Sxl alternative splicing, which determines female physiognomy	[38]
Heat shock response	Hsp105	Under heat shock stress, m^6^A is preferentially deposited at the 5’UTR of new stress-inducible transcripts, such as Hsp105 (HSPH1), and enhances cap-independent translation initiation	[28]
Somatic cell reprogramming and pluripotency of ESCs	Nanog, Sox2, Klf4 and c-Myc	High m^6^A modification levels accelerate mRNA degradation of these genes, which damages ESC self-renewal and somatic cell reprogramming	[37]

#### m^6^A modulates spermatogenesis

The process by which diploid spermatogonial stem cells (SSCs) produce haploid spermatozoa is called spermatogenesis [35]. m^6^A is reportedly present on the key regulatory factors of SSCs/progenitor cells, such as *Plzf, Id4, Dnmt3b,* and *Sohlh2,* which control the timing of transcript translation to coordinate normal protein generation, and this modification is essential for mammalian spermatogenesis [39]. m^6^A deletion resulted in the dysregulation of proliferation and differentiation factors of SSC/progenitor cells and SSC depletion [39].

#### m^6^A influences T cell homeostasis

Peripheral T cells are subject to complex and rigorous regulation, and the interleukin 7(*IL-7)/signal transducer and activator of transcription 5(STAT5)* signal axis is highly significant for maintaining naive T cell homeostasis and survival [36]. Decreased levels of m^6^A modification on the mRNAs of suppressor of cytokine signalling (*SOCS*) family genes have been reported to slow mRNA decay and increase protein expression levels (*SOCS1, SOCS3* and *CISH*) in naive T cells [40]. Overexpression of *SOCS1, SOCS3* and *CISH* leads to the inhibition of the downstream signal *IL-7/STAT5*, preventing naive T cell proliferation and differentiation while maintaining T cell survival [40]. Thus, m^6^A modifications are known to play an important role in T cell homeostasis.

#### m^6^A is involved in Drosophila sex determination

Mammalian m^6^A enzyme complexes include including WTAP, METTL14, RBM15, KIAA1429 and ZC3H13 [12–15]. The corresponding m^6^A methylation enzyme analogues in *Drosophila* include inducer of meiosis 4 (Ime4), karyogamy protein 4 (KAR4), female-lethal(2)d(Fl(2)d) and virilizer(Vir) [11, 12, 41–43]. The m^6^A reader protein YT521-B has been reported to read m^6^A modifications on *Sxl* to promote *Sxl* alternative splicing, which determines female physiognomy [38]. The ability of YT521-B to read m^6^A explains the importance of this modification in *Drosophila* sex determination through the selective splicing of *Sxl* [38].

#### High levels of m^6^A during the heat shock response

The heat shock response is a complex cellular reaction that causes significant changes in protein translation, folding and degradation, thereby mitigating toxic reactions caused by protein misfolding [44]. m^6^A and the heat shock response are linked because m^6^A is the most abundant mRNA post-transcriptional modification. A new report has revealed that m^6^A is preferentially deposited on the 5’UTR of new stress-inducible transcripts, such as *Hsp105 (HSPH1)*, under heat shock stress, and that increased levels of m^6^A modification at the 5’UTR can enhance cap-independent translation initiation [28]. Thus, the mechanistic connection between 5’UTR methylation and cap-independent translation reveals links between the heat shock response and m^6^A [28].

#### m^6^A influences somatic cell reprogramming and maintains the pluripotency of embryonic stem cells (ESCs)

Epigenetic and epitranscriptomic networks play important roles in somatic cell reprogramming and the maintenance of ESC pluripotency [37]. A new study has revealed that zinc finger protein 217 (*ZFP217*) activates the transcription of key pluripotency genes and modulates m^6^A deposition on their transcripts [37]. *ZFP217* depletion globally enhances m^6^A modification on *Nanog, Sox2, Klf4,* and *c-Myc* mRNAs to accelerate their degradation, thus damaging ESC self-renewal and somatic cell reprogramming [37]. This finding represents strong evidence of the close relationships between m^6^A and somatic cell reprogramming and the maintenance of ESC pluripotency.

### Aberrant m^6^*A* modification contributes to diversified tumours

**Fig. 2:**
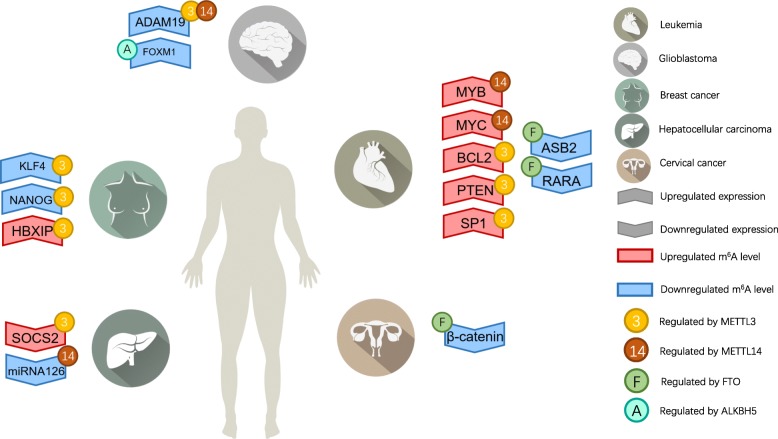
m^6^A modification functions as a ‘dual-edged sword’ in tumor progression. In AML, aberrant FTO, METTL14 and METTL3 lead to aberrant expression of the *ASB2, RARA, MYC, MYB, BCL2, SP1* and *PTEN* genes through m^6^A modification, ultimately promoting tumorigenesis. In GSCs, aberrant METTL3, METTL14 and ALKBH5 lead to the aberrant expression of *ADAM19* and *FOXM1* through m^6^A modifications, ultimately promoting tumorigenesis. In HCC, aberrant METTL3 and METTL14 lead to the aberrant expression of *SOCS2* and *miR126* through m^6^A modifications, ultimately promoting tumorigenesis. In BCSCs, aberrant METTL3 leads to the aberrant expression of *KLF4, NANOG* and *HBXIP* through m^6^A modifications, ultimately promoting tumorigenesis. In cervical cancer, aberrant FTO leads to the aberrant expression of β-catenin

Given the important role of RNA m^6^A modification in regulating gene expression and various biological processes [2], it is reasonable to speculate that aberrant m^6^A modification plays an important role in human carcinogenesis. However, knowledge of the mechanistic link between m^6^A and human carcinogenesis is rather limited. While investigations addressing this issue are still at an early stage, efforts are underway to explore the biological impacts of m^6^A modifications in cancer. We will summarize recent reports describing our understanding of the biological functions and underlying molecular mechanisms of m^6^A regulatory proteins in various types of cancer and explore new options for cancer treatment (Fig. 2 and Table 2).

**Table 2:** Aberrant m^6^A modification plays an important role in tumorigenesis

Tumour Type	Gene involved	Gene function	Description	reference
Acute myeloid leukaemia	ASB2 RARA	Anti-oncogene	Elevated FTO leads to low levels of m^6^A on ASB2 and RARA at UTRs, which reduces the mRNA and protein levels of these two genes	[47]
	MYB MYC	Oncogene	METTL14 enhances m^6^A modification of MYB and MYC, which in turn leads to overexpression of MYB and MYC	[48]
	BCL2 PTEN	Oncogene	Increased METTL3 in AML enhances m^6^A modification of BCL2 and PTEN, which leads to overexpression of BCL2 and PTEN	[50]
	SP1	Oncogene	METTL3 binds to the promoter region of sp1 and enhances m^6^A modification and gene expression	[51]
Hepatocellular carcinoma	SOCS2	Anti-oncogene	High expression of METTL3 in human HCC leads to high m^6^A levels on SOCS2, causing the rapid degradation of SOCS2	[60]
	microRNA126	Anti-oncogene	Decreased METTL14 reduces m^6^A modification levels and the expression of microRNA126	[61]
Glioblastoma stem cells	ADAM19	Oncogene	Low levels of METTL3 and METTL14 decrease m^6^A modification of ADAM19, which enhances the expression of ADAM19	[69]
	FOXM1	Oncogene	High levels of ALKBH5 decrease m^6^A modification levels of FOXM1 and enhance the expression of FOXM1, which ultimately causes glioblastoma	[70]
Breast cancer	KLF4 NANOG	Oncogene	ZNF217 interacts with METTL3 and inhibits the m^6^A methylation of KLF4 and NANOG, which ultimately leads to high expression of KLF4 and NANOG	[74]
	HBXIP	Oncogene	High levels of METTL3 enhance m^6^A modification of HBXIP, which accelerates HBXIP expression	[75]
	MAGI3	Anti-oncogene	High levels of m^6^A modification in the large internal exon of MAGI3 promote the occurrence of breast cancer	[31]
Cervical cancer	β-catenin	Anti-oncogene	Upregulated FTO represses m^6^A modification of β-catenin and induces chemoradiotherapy resistance	[79]

#### Connection between m^6^A and acute myeloid leukaemia (AML)

AML is one of the most common haematopoietic malignancies and is associated with a high mortality and distinct genetic and molecular abnormalities that lead to unsatisfactory therapeutic effects [45, 46]. Only a small proportion of patients survive for more than 5 years with standard chemotherapies [45, 46]. Therefore, it is urgent and necessary to explore new treatments for AML based on a deep understanding of the mechanisms of AML occurrence and development.

FTO, an obesity risk-associated gene and the first m^6^A eraser to be identified, has been reported to play an important oncogenic role in haematopoietic cell transformation and AML [17, 47]. FTO levels in certain subtypes of AML (e.g., t(11q23)/MLL-rearranged, t(15;17), FLT3-ITD, and/or NPM1-mutated) are abnormally elevated, which leads to the downregulation of m^6^A levels on the UTRs of *ASB2* and *RARA* [47]. These low levels of m^6^A reduce the mRNA and protein levels of these two genes [47]. FTO is not the only demethylase with a link with AML; METTL14 also plays an important oncogenic role in this disease by regulating its mRNA targets (e.g., *MYB, MYC*) through m^6^A modifications, which in turn leads to enhanced *MYB* and *MYC* expression and ultimately blocks myeloid differentiation [48]. Additionally, a new report has shown that FTO promotes the stability of *MYC* mRNA by inhibiting YTHDF2-mediated RNA decay, which is attributed to decreased m^6^A abundance on the 5′-terminal and internal exons of *MYC* mRNA, indicating that m^6^A modifications on different regions of the same mRNA transcript (e.g., *MYC*) lead to distinct fates [48, 49].

Furthermore, it has been reported that the presence of increased METTL3 levels in AML leads to higher m^6^A methylation levels of *BCL2* and *PTEN* and promotes the translation of *BCL2* and *PTEN* mRNA, which ultimately leads to tumour formation [50]. Recently, another study revealed that METTL3 binds to the promoter region of *SP1* with the aid of transcription factor *CEBPZ*, which enhances m^6^A modification of *SP1*, strengthens *SP1* gene expression, and ultimately leads to myeloid leukaemia [51]. In general, changes in m^6^A modification levels on *ASB2, RARA, MYC, MYB, BCL2*, *SP1*and *PTEN* eventually lead to the occurrence of AML [47–50].

#### Aberrant m^6^A in hepatocellular carcinoma (HCC)

HCC is a major type of primary liver cancer, accounting for the 5th highest incidence of malignant tumours worldwide and causing more than 700,000 annual deaths [52]. The prevalence of hepatitis B and C viruses in Asia is the main cause of the high incidence of liver cancer in Asia [52]. Effective interventions are lacking, resulting in high HCC mortality due to metastasis and recurrence; thus, a deeper understanding of the molecular mechanism underlying the occurrence and development of HCC is required. Growing evidence suggests that the occurrence of liver cancer is a multistep process involving complex interactions between genetics, epigenetics and transcriptional changes [53]. Previous studies have shown that DNA hypermethylation occurs on CpG islands of the promoter regions of tumour suppressor genes, such as *DLC1, TFPI-2, CDKN2A*, and *PTEN*, ultimately affecting tumorigenesis and development [54–56]. It was reported that high levels of the histone methyltransferases *EZH2, SUV39H1, SETDB1* and *G9a* promote the development and metastasis of HCC via epigenetic silencing of critical tumour suppressor genes [57–59]. In general, abnormal epigenetic modifications may be important factors in the development of liver cancer.

Many articles note that the development of liver cancer is associated with abnormal m^6^A modifications [60, 61]. The high expression of METTL3 in human HCC reportedly leads to increased m^6^A modification levels on the tumour suppressor *SOCS2* [60]. Excessive m^6^A chemical modification of *SOCS2* is read by YTHDF2, which accelerates the degradation of *SOCS2* and eventually leads to the occurrence of HCC [60]. Another report has indicated that decreased *METTL14* expression reduces m^6^A modification levels and the expression of *microRNA126 (miR126*) [61]. Low m^6^A modification levels on *miR126* are recognized by *DGCR8* and may ultimately promote liver carcinogenesis [61]. These articles strongly suggest that abnormal m^6^A modification plays an important role in the occurrence and development of HCC.

#### The foundation role of m^6^A in glioblastoma stem cells (GSCs)

Glioblastomas are primary brain tumours with a high degree of malignancy [62]. The median survival time after diagnosis is usually less than 15 months, even if diagnosis is combined with surgical resection, radiotherapy and chemotherapy [62, 63]. GSCs are a group of tumour stem cells with the ability to promote tumour growth and invasion, showing strong resistance to radiotherapy and chemotherapy; these characteristics are the main reasons for the poor prognosis of glioblastoma [64–67]. Therefore, the issue of stem cell resistance should be explored and addressed to improve therapeutic approaches to glioblastoma [66, 68]. RNA epigenetics has become a rapidly developing field in biology and may be valuable for informing glioblastoma treatment.

A study showed that low levels of METTL3 or METTL14, key components of the RNA methyltransferase complex, lead to decreased m^6^A modification levels on *ADAM19* and the enhanced expression of *ADAM19* in GSCs, ultimately causing glioblastoma [69]. Low m^6^A modification levels on *ADAM19* and high mRNA expression levels of *ADAM19* may represent a promising target for anti-glioblastoma therapy [69]. Another study revealed that aberrant m^6^A modifications caused by high levels of ALKBH5, an m^6^A demethylase, are an obvious physiological abnormality of GSCs [70]. Decreased m^6^A modification levels of *FOXM1* result in enhanced *FOXM1* expression levels, which ultimately cause glioblastoma [70]. Aberrant m^6^A modifications caused by different mechanisms in GSCs strongly suggest that this modification is related to the occurrence and development of glioblastoma.

#### Abnormal m^6^A modification in breast cancer

Among all malignant tumours in women, breast cancer has the highest incidence and leads to the highest number of deaths [71, 72]. Although the therapeutic outcomes of early-stage breast cancer are relatively good, treatments for metastasis are not effective [73]. Given the high recurrence and mortality rate, the molecular mechanisms that regulate breast cancer phenotypes need to be carefully delineated and studied to design more effective therapies.

In breast cancer stem cells **(**BCSCs), *ZNF217* has been reported to interact with METTL3 and inhibit the m^6^A methylation of *KLF4* and *NANOG*, which ultimately leads to high expression of *KLF4* and *NANOG*, thus promoting tumorigenesis [74]. Another report indicated that high m^6^A modification levels on hepatitis B X-interacting protein (*HBXIP*) and the overexpression of *HBXIP* caused by high METTL3 levels accelerate the proliferation of BCSCs [75]. In addition, a study revealed that high levels of m^6^A modification on *MAGI3* lead to premature polyadenylation, switching its functional role from a tumour suppressor gene to a dominant-negative oncogene and ultimately promoting tumorigenesis of breast cancer [31]. The aberrant m^6^A modifications caused by different mechanisms in breast cancer prove that this modification is related to the occurrence and development of breast cancer.

#### Aberrant m^6^A modification in cervical cancer

Cervical cancer is one of the most common and destructive gynaecological malignancies [76]. Chemoradiotherapy is the major therapy used to treat cervical squamous cell carcinoma [77]. However, chemoradiotherapy resistance is the major cause of treatment failure. Therefore, it is necessary to further understand the molecular mechanisms underlying chemoradiotherapy resistance and explore novel therapeutic treatments for cervical squamous cell carcinoma (CSCC) [78].

In CSCC, the expression of FTO is significantly higher than that in normal tissues, resulting in lower levels of m^6^A modification in *β-catenin*, which causes decreased expression of *β-catenin* and chemoradiotherapy resistance. The discovery of this mechanism suggests that *MA2*, a novel small molecular inhibitor of FTO, may increase the chemoradiotherapy sensitivity of CSCC [79].

#### METTL3 promotes the translation of oncogenes in human lung cancer

Lung cancer is one of the most common malignant tumours in humans, causing many deaths every year [80, 81]. The 5-year survival rate of lung cancer patients is still very low despite continuous improvement and progress in the diagnosis and treatment of lung cancer [82, 83]. Non-small-cell lung carcinoma (NSCLC) accounts for 85% of all pathological types of lung cancer [82, 83]. Our attention should be focused on the abnormal molecular biological characteristics of NSCLC to find an effective treatment.

Many articles have reported that abnormal m^6^A modifications ultimately affect tumour development. However, one report indicated that in lung cancer, METTL3 associates with translation machinery and enhances the translation of target mRNA (*RGFR* and *TAZ*) independent of its methyltransferase activity [84]. Another report also indicated that *miR-33a* prohibits NSCLC cell proliferation by targeting METTL3, which suggests that *miR-33a* may be a potential molecule for therapy [85]. Moreover, post-translational modification of *METTL3* has been revealed. For example, METTL3 is modified by SUMO1, and SUMOylation of METTL3 decreases m^6^A levels on mRNAs, which ultimately promotes the development of NSCLC [86].

#### m^6^A leads to the acceleration of tumour formation

In AML, upregulated m^6^A modification on *MYB, MYC, BCL2, PTEN* and *SP1* results in enhancement of the binding capability and translational efficiency of onco-RNA and ribosomes, leading to tumorigenesis [47–50]. Moreover, in hepatocellular carcinoma, the excessive m^6^A modification of the *SOCS2* tumour suppressor gene reduces mRNA stability and accelerates its degradation, which causes tumour progression [60]. In addition, in breast cancer, upregulated m^6^A modification of HBXIP and MAGI3 results in tumour formation [31]. It is worth noting that excessive modification of *MAGI3* leads to premature polyadenylation, switching its functional role from that of a tumour suppressor gene to a dominant-negative oncogene, ultimately promoting tumorigenesis [31]. In summary, RNA methylation triggers certain alterations to tumour-specific mRNA behaviour and results in changes in onco-protein expression and biologic activity, thereby accelerating the tumour progression.

#### m^6^A contributes to the inhibition of tumour development

In contrast, the aberrant decreased m^6^A modification levels on target loci can also disrupt normal RNA functions, which in turn restores normal m^6^A levels on these targets and ideally suppresses tumour formation. The FTO-mediated downregulation of m^6^A modification levels on ASB2 and RARA leads to the downregulation of these anti-oncogenes via RNA and protein degradation, leading to the promotion of tumorigenesis [47]. In hepatocellular carcinoma, the decreased m^6^A modification levels on microRNA126 influence its function as a ceRNA and disrupt its regulation of binding capability, thereby triggering the acceleration of tumour development [61].

Similarly, in GSCs, a long non-coding RNA *FOXM1-AS* directly binds to FOXM1 mRNA, enhancing the interaction between ALKBH5 and FOXM1 nascent transcripts and giving rise to reduced m^6^A modification levels and the overexpression of this oncogene [70]. Additionally, decreased m^6^A modification levels enhance the RNA stability of KLF4 and NANOG and ultimately contribute to tumour formation [74]. In summary, it has been revealed that decreased RNA methylation may participate in tumorigenesis. Therefore, a novel therapeutic strategy may involve tumour suppression via enhanced m^6^A modification to balance the transcription of these genes.

## Conclusion

In summary, an increasing number of studies has shown that aberrant m^6^A modification is closely related to tumorigenesis, including AML, HCC, GSCs, breast cancer, cervical cancer and lung cancer [50, 60, 70, 75, 79]. Moreover, numerous genes modified by m^6^A have been revealed to play regulatory roles in tumour formation, such as BCL2, PTEN, SOCS2, FOXM1 and HBXIP [50, 60, 70, 75, 79]. In conclusion, m^6^A modification is a double-edged sword, over-modification of a target gene by m^6^A could result in altered RNA splicing and translational capability, leading to the acceleration of cancer formation, whereas the lack of m^6^A modification at other loci may also contribute to tumorigenesis.

Abnormal levels of m^6^A methylation may give rise to tumour progression. However, we should not ignore the notion that RNA methylation enzymes influence tumorigenesis in an m^6^A-independent manner. For example, in lung cancer, *METTL3* directly associates with translation machinery and enhances the translation of target mRNA (*RGFR* and *TAZ*) independent of its methyltransferase activity [84].

This review updates our knowledge of the aberrant m^6^A methylation of diverse target loci and discusses its impact on tumour formation. Aberrant levels of m^6^A modification, such as increased or decreased levels, may alter RNA splicing, RNA-coding capability or onco- or tumour suppressor genes. To discover novel tumour therapies based on the evaluation of m^6^A modifications, it should be noted that m^6^A functions as a dual-edged weapon; thus, restoring ideal levels of m^6^A (rather than simply over-supplementing or over-silencing) holds great significance.

## Abbreviations

3’UTRs: 3′ untranslated regions
*ADAM19*: A disintegrin and metallopeptidase domain 19
ALKBH5: AlkB homologue 5
AML: Acute myeloid leukaemia
*ASB2*: Ankyrin repeat and SOCS box containing 2
A-to-I: Adenosine-to-inosine
*BCL2*: B cell leukaemia 2
BCSCs: Breast cancer stem cells
*CDKN2A*: Cyclin dependent kinase inhibitor 2A
*CISH*: Cytokine inducible SH2 containing protein
CSCC: Cervical squamous cell carcinoma
*Dnmt3b*: DNA methyltransferase 3B
ESCs: Embryonic stem cells
*EZH2*: Enhancer of zeste 2 polycomb repressive complex 2 subunit
Fl(2)d: Female-lethal(2)d
FMR1: Fragile X mental retardation 1
*FOXM1*: Forkhead box M1
FTO: Fat mass and obesity-associated protein
GSCs: Glioblastoma stem cells
*HBXIP*: Hepatitis B X-interacting protein
HCC: Hepatocellular carcinoma
HNRNP: Heterogeneous nuclear ribonucleoprotein
*Id4*: Inhibitor of DNA binding 4
IGF2BPs: Insulin-like growth factor 2 mRNA-binding proteins
*IL-7*: Interleukin 7
Ime4: Inducer of meiosis 4
KAR4: Karyogamy protein 4
*Klf4*: Kruppel like factor 4
*KLF4*: Kruppel like factor 4
LRPPRC: Leucine rich pentatricopeptide repeat containing
m^6^A: N6-methyladenosine
*MAGI3*: Membrane associated guanylate kinase
*MAGI3*: Membrane associated guanylate kinase, WW and PDZ
*MALAT1*: Metastasis-associated lung adenocarcinoma transcript 1
*MAT2A*: Methionine adenosyl transferase 2A
METTL14: Methyltransferase-like 14
METTL16: Methyltransferase-like protein 16
METTL3: Methyltransferase-like 3
*MYB*: Myeloblastosis oncogene
*MYC*: Myelocytomatosis oncogene
*NANOG*: Nanog homeobox
NSCLC: Non-small-cell lung carcinoma
*Plzf*: Promyelocytic leukaemia zinc finger
*PTEN*: Phosphatase and tensin homolog
*RARA*: Retinoic acid receptor alpha
RBM15: RNA binding motif protein 15
SAM: S-adenosylmethionine
*SETDB*: SET domain bifurcated 1
*SOCS2*: Suppressor of cytokine signaling 2
*Sohlh2*: Spermatogenesis and oogenesis specific basic helix-loop-helix 2
*Sox2*: Sex determining region Y box 2
SSCs: Spermatogonial stem cells
*STAT5*: Activator of transcription 5
SUMO1: Small ubiquitin-like modifier 1
*SUV39H1*: Suppressor of variegation 3–9 homolog 1
*Sxl*: Sex lethal
*TFPI-2*: Tissue factor pathway inhibitor 2
WTAP: Wilms tumour 1-associated protein
YTH: YT521-B homology
ZC3H13: Zinc finger CCCH-type containing 13
*ZFP217*: Zinc finger protein 217
*ZNF217*: Zinc finger protein 217
